# Radar Human Activity Recognition with an Attention-Based Deep Learning Network

**DOI:** 10.3390/s23063185

**Published:** 2023-03-16

**Authors:** Sha Huan, Limei Wu, Man Zhang, Zhaoyue Wang, Chao Yang

**Affiliations:** School of Electronics and Communication Engineering, Guangzhou University, Guangzhou 510006, China

**Keywords:** human activity recognition (HAR), attention mechanism

## Abstract

Radar-based human activity recognition (HAR) provides a non-contact method for many scenarios, such as human–computer interaction, smart security, and advanced surveillance with privacy protection. Feeding radar-preprocessed micro-Doppler signals into a deep learning (DL) network is a promising approach for HAR. Conventional DL algorithms can achieve high performance in terms of accuracy, but the complex network structure causes difficulty for their real-time embedded application. In this study, an efficient network with an attention mechanism is proposed. This network decouples the Doppler and temporal features of radar preprocessed signals according to the feature representation of human activity in the time–frequency domain. The Doppler feature representation is obtained in sequence using the one-dimensional convolutional neural network (1D CNN) following the sliding window. Then, HAR is realized by inputting the Doppler features into the attention-mechanism-based long short-term memory (LSTM) as a time sequence. Moreover, the activity features are effectively enhanced using the averaged cancellation method, which improves the clutter suppression effect under the micro-motion conditions. Compared with the traditional moving target indicator (MTI), the recognition accuracy is improved by about 3.7%. Experiments based on two human activity datasets confirm the superiority of our method compared to traditional methods in terms of expressiveness and computational efficiency. Specifically, our method achieves an accuracy close to 96.9% on both datasets and has a more lightweight network structure compared to algorithms with similar recognition accuracy. The method proposed in this article has great potential for real-time embedded applications of HAR.

## 1. Introduction

Owing to the increasing demand for intelligent health monitoring, comprehensive traffic management, smart security, and advanced surveillance systems, human activity recognition (HAR) is being promoted. These application scenarios place high requirements on the speed and accuracy of the HAR method. As the two main non-wearable sensing modalities, video and radar can meet the above requirements. Compared to the ubiquitous availability of video sensors, radar-based HAR is characterized by a number of fundamental relative merits, including robustness to lighting conditions and visual obstructions. Moreover, a radar sensor can preserve the visual privacy of the identified human subjects.

Radar-based HAR mainly constitutes the analysis of the human activity characteristics embedded in the radar echoes. In addition to the essential range and velocity information that can be extracted from the main reflection of a human target, radar echoes contain the micro-motion information of the different moving parts of the human body. All the movements can be characterized by the Doppler signal of the human target. The superposition of all these Doppler signals constitutes the micro-Doppler feature (MDF), which is one of the most promising features for HAR. The offset of target echo in the Doppler frequency domain can also play an important role in multiple input multiple output (MIMO) radar SAR imaging. In [1], high-resolution imaging is achieved by adopting the Doppler-division multiplexing (DDM) technique for MIMO channel separation and combining it with a single-channel BP algorithm and multi-channel synthesis.

Aimed at MDF-based HAR, a coherent integration scheme, such as the short-time Fourier transform (STFT) or the Wigner–Ville distribution, is usually applied to obtain the time-frequency (TF) spectrogram. Thereafter, HAR can be realized by extracting the time-varying features of the micro-Doppler signal in the spectrogram. Traditional methods for MDF categorization are based on manually defined spectral description words, which comprise several statistical features, including the bandwidth of the Doppler signal, the torso Doppler frequency, and the normalized standard deviation of the Doppler signal strength [2]. Fairchild et al. [3] used the empirical mode decomposition to produce a unique feature vector to represent the MDF. Then, HAR is realized in conjunction with a support vector machine (SVM) classifier. As a supervised learning algorithm, SVM is usually suitable for large sample sizes. For limited sample sizes in unsupervised learning, Sharifi [4] used a relevance vector machine (RVM) to extract flood maps and achieved a classification performance of 89%. However, the accuracy and efficiency of the abovementioned methods are limited by classification complexity.

Recently, deep learning (DL) has received significant attention for its superior recognition performance. Radar-based HAR has also experienced an influx of DL research. These approaches to DL can be roughly divided into the following three classes: a convolutional neural network (CNN); a recurrent neural network (RNN); and a hybrid network. These methods automatically extract the features of the samples using supervised learning, overcoming the deficiencies of traditional models in feature extraction.

Typical CNN and numerous deep variants of CNN have been proposed to enhance radar-based HAR performance. Kim et al. [5] first applied deep CNN to HAR and achieved impressive classification results. The literature [6,7] employed a fusion of multi-dimensional CNN to improve recognition accuracy. Moreover, some researchers have attempted to reduce the computational complexity and improve the inference speed using lightweight CNN. Zhu et al. [8] proposed a lightweight CNN, which gives a high recognition accuracy but requires only a few parameters. Despite these considerable advantages, the interpretation of MDF as two-dimensional (2D) images focuses on the spatial correlation features between the pixels. Thus, the extracted features are highly redundant, which affords a complex network with numerous parameters and high hardware requirements. In fact, the activity features in TF are better reflected in the temporal correlation of Doppler sequences rather than in the spatial structure of the 2D image.

A temporal network model employing a recurrent neural network can extract the temporal correlation features between data sequences. The literature [9,10,11] employed RNN, long short-term memory (LSTM), and bi-directional LSTM (Bi-LSTM) to realize HAR, respectively. They can all achieve good recognition results. In addition, Shrestha et al. [12] combined LSTM with Bi-LSTM to implement HAR. Jiang et al. [13] used stack-LSTM to implement human activity classification. They both can achieve over 90% average accuracy, but they have a very large number of network parameters. This means that such a complex network model is difficult to apply to embedded applications that are limited by hardware resources and computing power.

Hybrid networks, such as CNN-LSTM [14,15,16,17,18,19], can achieve enhanced performance compared to individual networks as they combine the expertise of the constituent networks. The hybrid structure can fully exploit the space–time characteristics of input data and improve the accuracy of recognition. The above network typically feeds the hidden state output from the last time step ($h_{T}$) or the hidden state output from all time steps of the LSTM to the classifier for output. In the former case, $h_{T}$ only highlights the feature representation within the current time period and the memory effect is limited for long sequences. In the latter case, irrelevant temporal features may also be introduced into the output. Both of these processing methods can lead to limited recognition performance of the network.

Attention is a cognitive process of selectively concentrating on the important things while ignoring others in psychology. Researchers have applied this idea in many tasks, such as semantic analysis [20] and image segmentation [21]. The attention module is usually not used alone, it can be added to different neural networks to improve the performance of the networks. The literature [22,23,24,25,26] introduced the attention mechanism to the residual network (ResNet101), convolutional autoencoder (CAE), CNN, LSTM, and Bi-LSTM, respectively. The networks with the attention mechanism converge faster than the networks without the attention mechanism, and their recognition accuracy is higher than the network without the attention mechanism. In addition, the attention module is quite flexible; it can be added anywhere in the network, such as [27] who used the attention modules before the proposed Multi-RNN. Attention usually eliminates the vanishing gradient problem as it provides direct connections between all the time step data. Moreover, the distribution of attention weights provides an intuitive insight into the activity of the training model.

Most of the above studies have focused on improving the accuracy of HAR while ignoring the issue of limited computational and storage resources on embedded devices. That is to say, there is relatively little research on lightweight networks in the field of HAR. Therefore, to achieve an efficient recognition network with a lightweight structure for HAR, researchers need to consider various factors such as the generation mechanism of micro-Doppler signals mapped from human activity, pre-process the characteristics of target echoes. Furthermore, researchers also need to consider the data format at the network input and design a reasonable network structure based on the distribution characteristics of micro-Doppler signals, in order to expand HAR to some embedded applications.

**Figure 1:**
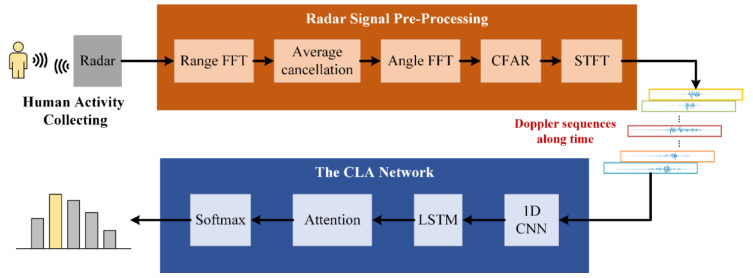
Proposed HAR system.

In this paper, we proposed a solution for HAR. Figure 1 displays the block diagram of the proposed system. The system consists of three parts: human activity collecting; radar signal pre-processing; and the CLA network. In this study, the analysis is performed based on the experimental data collected by a millimeter-wave band radar, which advantageously has a high resolution, high detection accuracy, small size, and low cost. In the radar signal pre-processing stage, we utilized fast Fourier transform (FFT) to compress the distance and angle of the target and implemented the average cancellation algorithm to suppress fixed clutter. Additionally, we used the constant false alarm rate (CFAR) for target detection, which improved the availability of data. Finally, STFT was employed to obtain Doppler sequences of human activity data. In this study, a hybrid DL model based on the attention mechanism CLA (CNN–LSTM–Attention) is proposed. The proposed model has a relatively light framework and supports advanced features. One-dimensional (1D) CNN is adopted herein for spectral feature acquisition of Doppler sequences. Without the completion of the 2D micro-Doppler map (MDM) after STFT sliding window processing, the spectral vector generated by each window can be fed into the 1D CNN. This method effectively avoids the high redundancy of 2D CNN feature extraction and reduces time consumption. Then, LSTM extracts the temporal dependence of the time-varying frequency features under different time steps, obtaining the global temporal information related to human activities. Finally, the attention module utilizes the attention values to reassign the weights of the time-varying frequency features and integrates the frequency and time dimension characteristics for recognition, which effectively enhances feature representation. 

This article proposes an efficient and lightweight HAR scheme based on the attention mechanism, which focuses on two aspects: radar signal pre-processing and the CLA network. The introduced average cancellation algorithm in the radar signal processing part can enhance the features of human activity, thus improving the recognition performance of HAR. The proposed CLA adopts a 1D processing network based on the attention mechanism, which fully exploits the Doppler and timing characteristics of the target activities reflected in the radar signal. The experimental results show that our proposed method can not only achieve high classification accuracy but also has a lighter network structure compared to traditional algorithms with similar recognition accuracy. This has great potential for resource- and storage-constrained embedded applications.

The contributions of our work are summarized as follows:

(1) We proposed a hybrid network that incorporates the attention module. The network decouples the Doppler and temporal features of human activity in MDM. Based on 1D CNN to extract the Doppler features of time sequences, the network can be more lightweight. Meanwhile, the attention-based LSTM can extract important time features between the Doppler feature sequences, thus enhancing the network’s ability to capture key features. The experimental results show that the network proposed herein can achieve the best accuracy and relatively low complexity.

(2) In the raw radar signal processing part, the average cancellation method is utilized to suppress the fixed clutter interference, which has been proven to be more suitable for micro-Doppler analysis than the traditional moving target indicator (MTI). Moreover, it improves the recognition performance of HAR compared to signal processing without suppression.

(3) This study explores the optimal structure of the proposed network through a self-established dataset with five types of human behavior. A comparative analysis was conducted with some state-of-the-art HAR networks using a self-established dataset and a public dataset. The use of two different datasets ensures that the final results of the experiment are fairer and more reasonable. The results show that the proposed method in this paper achieves satisfactory accuracy in both datasets. 

This paper is organized as follows. Section 2 provides the details of the micro-Doppler signal process based on the FMCW radar signal model. Section 3 illustrates the structure of the CLA hybrid multi-network for HAR. Section 4 provides the experimental results of real data to verify the superiority of the proposed algorithm. Finally, Section 5 presents the conclusions.

## 2. Micro-Doppler Signal Processing

### 2.1. Signal Model in the FMCW Radar

**Figure 2:**
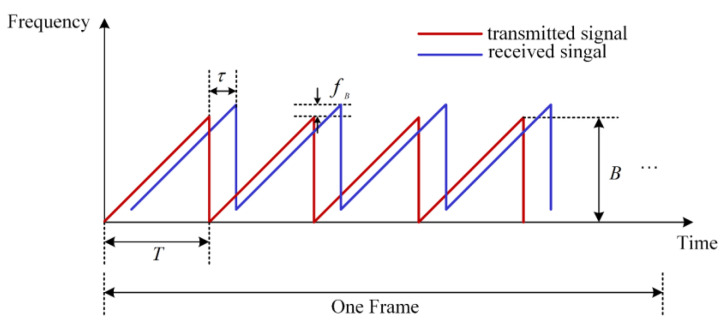
FMCW radar waveform in the TF domain.

Consecutive chirp signals are often used as the waveform of the FMCW radar. Figure 2 displays the waveform structure in the TF domain.

The radar transmits a series of chirp signals with a period of T. Each chirp can be expressed as follows:

(1) $$S_{T}\left(t\right)=\exp\left(j2\pi \left(f_{c}t+\frac{1}{2}\frac{B}{T}t^{2}\right)\right)$$

where $f_{c}$ is the carrier frequency, B is the signal bandwidth, and T is the duration of the chirp. Additionally, N chirp pulses form one frame.

Transmitted signals are reflected when they encounter the human body and environmental targets. Echoes and transmitted signals mix and yield an intermediate frequency (IF) signal with a lower frequency, which is conducive to digitization. The expression of the IF signal is:

(2) $$S_{IF}\left(t\right)=\exp\left(j2\pi \left(\left(\frac{2\frac{B}{T}R+2f_{c}V}{c}\right)t+\frac{2Rf_{c}}{c}\right)\right),$$

where R is the range between the radar and the target, V is the radial velocity of the target, and c denotes the speed of light. The target motion generates a Doppler shift $f_{D}=\frac{2f_{c}V}{c}$. When the target is a moving human body, the motion tendency of the torso constitutes the main part of the Doppler signal in the echo. The limb swing state, which constitutes the motion details, is reflected on both sides of the main Doppler signal in the spectrum. This is the micro-Doppler signature introduced by human motion in radar echoes.

Owing to its high operating frequency, the millimeter-wave radar not only has the advantage of high range resolution due to its large bandwidth but can also elucidate the detailed characteristics of limb movement. HAR mainly aims to sort the micro-Doppler information afforded by human motion.

### 2.2. Micro-Doppler Processing

**Figure 3:**
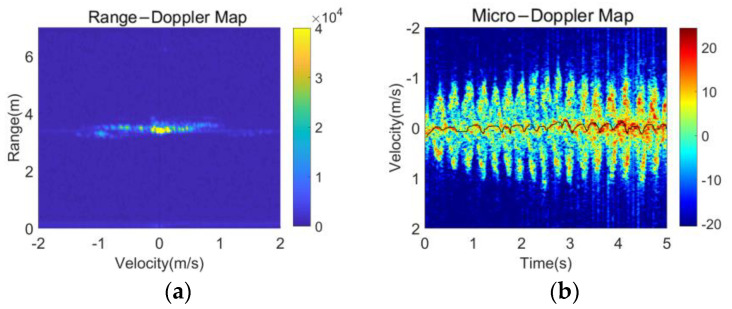
Different feature representation maps of human running: (**a**) RDM and (**b**) MDM.

Conventional range-Doppler (RD) compression can denote motion-induced Doppler expansion, but it loses the time-varying features of human activity, as shown in Figure 3a. Therefore, human motion patterns can hardly be specified, and different human activities can barely be distinguished only based on the RD features. TF processing is usually applied to preserve this time-varying information of motion in MDM. MDM can simultaneously present the Doppler features of the torso and limbs during human activity. In Figure 3b, the black curve represents the velocity trajectory of the torso, which exhibits only small fluctuations. MDFs corresponding to limb movements exhibit pronounced periodic variations, providing a high degree of feature discrimination.

In addition to accurately extracting motion information via TF processing, the algorithm needs to effectively eliminate the fixed clutter interference. Subsequently, the accuracy of activity recognition will be improved. The signal processing method used herein will be comprehensively introduced below.

**Figure 4:**
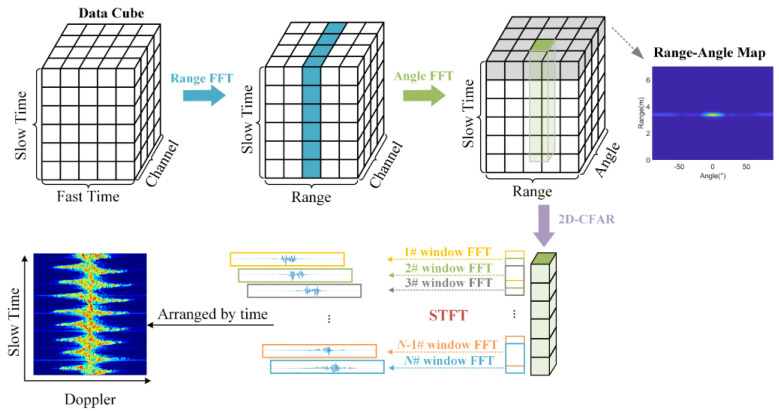
Micro-Doppler processing flow.

The digital IF data of the MMW radar can form a data cube according to three dimensions: fast time, slow time, and receive channel. By separately performing FFT in the fast time and space dimensions, the 2D compression result of the range-angle map (RAM) can be obtained along the slow time axis, as shown in Figure 4. Then, 2D-CFAR [28] is used to lock the bin at the location where the target is located. After locating the target bin, the sequence of target bins distributed in the slow time can be used to extract the time-varying behavioral features.

The TF diagram is the most used representation for analyzing the time-varying Doppler characteristics of human motion, and it contains Doppler information of the signal at different time periods. The TF diagram can be computed with STFT, which implements Fourier transform based on a sliding time window. The Doppler sequences generated by each sliding window segment are arranged based on the slow time to form the TF diagram. This diagram is the MDM of the target. Doppler signatures introduced by different behaviors are clearly seen in the MDM.

### 2.3. Static Clutter Suppression

In practical applications, the power of the micro-motion signal introduced by human activity is so small in the echo that it is easily covered by strong static clutter, such as the torso and background. When the MDM value is normalized, the existence and variation of the micro-Doppler signals are compressed due to the existence of strong static clutter. This compression impairs the MDF, affecting the subsequent feature extraction.

Therefore, to highlight the micro-motion characteristics on MDM, the average cancellation method is applied herein to suppress the static clutter instead of the traditional MTI [29]. Compared to MTI, the configuration of the average cancellation is characterized by a higher Q factor by introducing more transmission zeros in the transfer function. This allows the complete preservation of the micro-Doppler information while suppressing the static clutter. It can efficiently increase the signal-to-noise ratio of the micro-motion signal and improve the performance of the subsequent feature extraction.

The average cancellation method first captures the static clutter by summing the samples and dividing the sum by the number of samples. Then, the clutter signal is subtracted from the original signal. Subsequently, an enhanced micro-Doppler signal is obtained after static clutter subtraction. In this study, static clutter suppression is added after range compression. It can be expressed as:

(3) $$S_{RT\_AC}\left(m,n\right)=S_{RT}\left(m,n\right)-\frac{1}{N}\sum_{i=1}^{N}S_{RT}\left(m,i\right),$$

where m is the index in the range domain and n is the index along the slow time. The average length N is related to the sliding window length of STFT.

**Figure 5:**
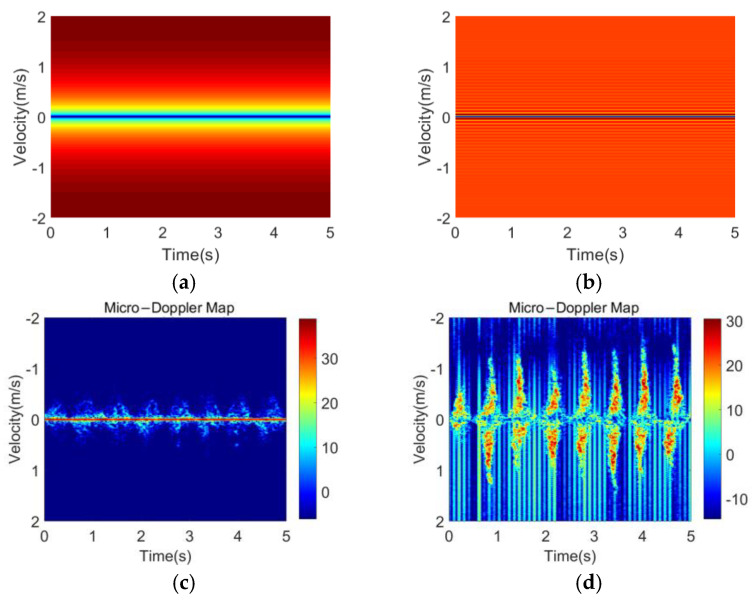

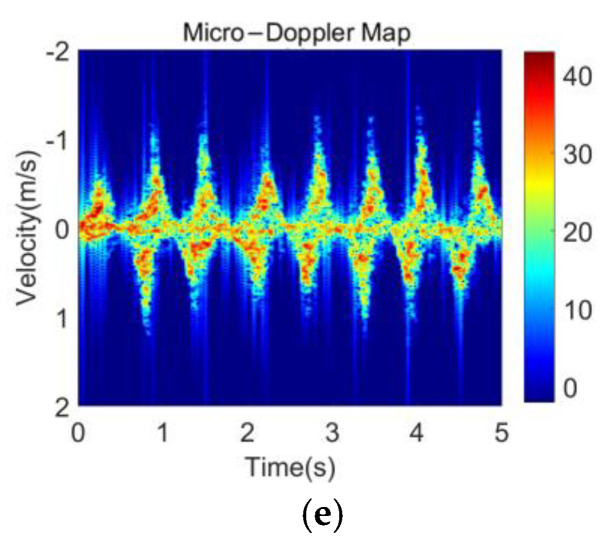
Comparison of the static clutter suppression methods in the walking state: (**a**) frequency response of MTI in the TF domain; (**b**) frequency response of the average cancellation in the TF domain; (**c**) MDM without static clutter suppression; (**d**) MDM after MTI; (**e**) MDM after the average cancellation.

**Figure 6:**
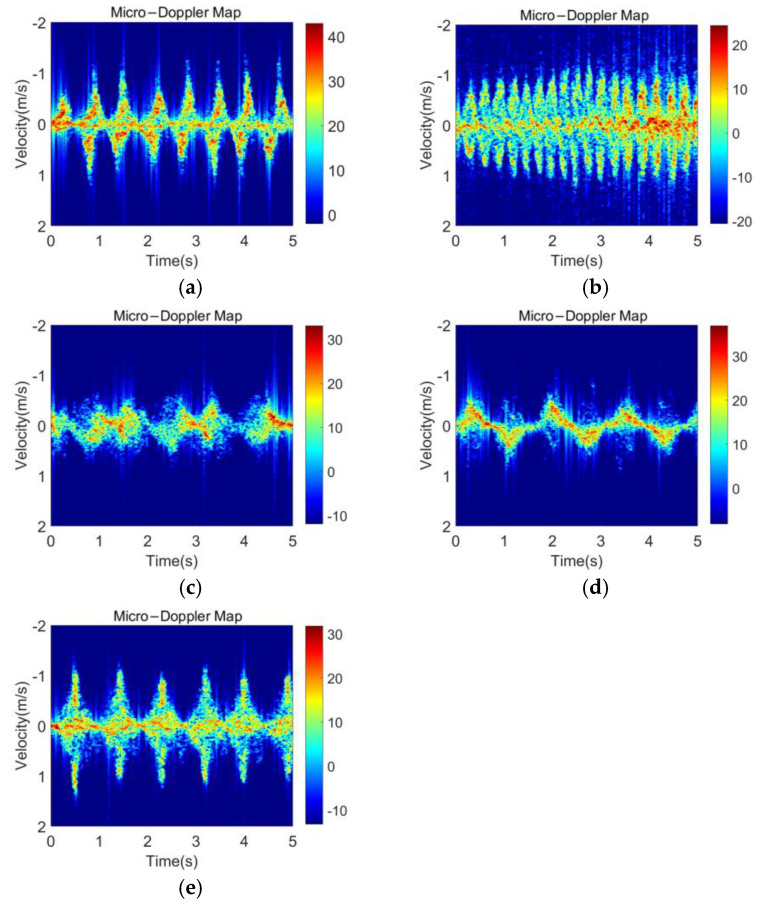
MDM after the average cancellation of five activities: (**a**) walking; (**b**) running; (**c**) standing up after squatting down; (**d**) bending; and (**e**) turning.

Figure 5 compares MDM with different clutter suppression algorithms in the walking state. Without the static clutter suppression, the main signal energy is concentrated in the 0-Doppler position and the micro-Doppler component is not obvious in MDM. Both MTI and average cancellation significantly increase the appearance of MDF on MDM by suppressing the signal component located at the 0-Doppler position. However, as shown in Figure 5a, the MTI method using phase difference can highlight the high-frequency component, but the wide transition band is not conducive to the complete preservation of the micro-Doppler information. The average cancellation has a narrow stopband at the 0-Doppler position and a flat passband; thus, it can obtain a relatively clean background while highlighting the MDF. Figure 6 shows the MDM after applying the average cancellation for walking, running, standing up after squatting down, bending, and turning.

## 3. CNN-LSTM-Attention Hybrid Multi-Network

Many studies on HAR have directly used MDM as a 2D image. However, MDM comprises multiple 1D Doppler spectrums splicing along the slow time segment-by-segment. The most significant disparity between the mechanism of MDM and 2D image is that MDM’s feature expression is the Doppler spectrum distribution change with time. Therefore, the direct processing of MDM as a 2D image not only cannot meet the real-time processing requirements but also ignores the unique continuous expression of human activity characteristics by MDM, resulting in unsatisfactory efficiency and accuracy.

**Figure 7:**
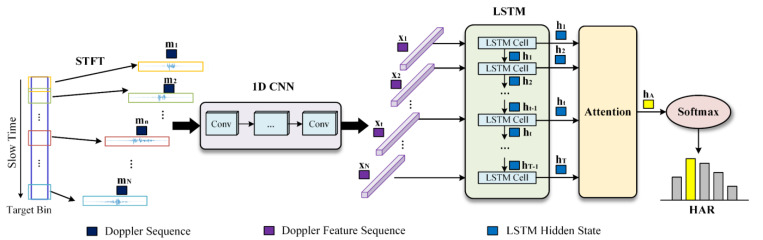
Proposed CLA network.

This study gives full consideration to the real-time performance and effectiveness of HAR. Three network structures are mixed to benefit from their respective advantages. A CLA hybrid multi-network is proposed to fully extract the MDF of human activity. The overall framework of the proposed network is shown in Figure 7. The 1D CNN extracts the target Doppler features within each time window through a compact network. Then, the LSTM network obtains the temporal correlation information between the Doppler features corresponding to the different windows. Finally, the weight allocation mechanism of the attention mechanism is applied to highlight the important features from the hidden states of LSTM.

Instead of waiting for the end of the window sliding operation of STFT, the Doppler distribution of each sliding window can be directly fed into the 1D CNN. Compared to multi-dimensional CNN, the 1D CNN better processes data in sequence with fewer network parameters, reducing the hybrid network complexity. The Doppler sequence output by each sliding window is defined as $m_{t}$, and the set of sequences generated by the entire STFT is $M=\left(m_{1},\cdots ,m_{T}\right)$.

The 1D CNN network employed herein comprises several convolutional layers, and the layers maintain the same feature scale using the same padding. The specific number of the convolutional layers and the convolutional kernels in each layer are discussed in Section 4 based on the accuracy and efficiency of the hybrid network. Furthermore, ReLU is adopted as the activation function in the 1D CNN convolution process. The 1D CNN extracts the Doppler feature of $m_{t}$ according to the sequence generation order. Then, the Doppler distribution of $m_{t}$ under the current time window is converted into a Doppler feature sequence $x_{t}$ through the convolution modules. The sequence set composed of $x_{t}$ preserves all the temporal correlation information between the Doppler feature sequences.

**Figure 8:**
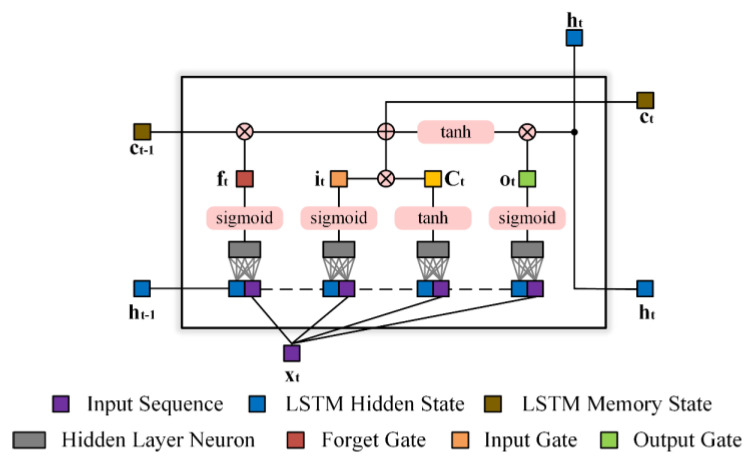
Internal network structure of the LSTM cell.

LSTM is used to process the temporal correlation features between the Doppler feature sequences. LSTM is an improved RNN network, which alleviates the problem of RNN gradient disappearance and captures long-time dependents. It controls the transmission state of information through a gate mechanism to retain the temporally pertinent information across the time steps. The internal network structure of an LSTM cell is shown in Figure 8.

In the proposed CLA network, the input sequence of the LSTM cell is $x_{t}$. The expression of the corresponding forget gate $f_{t}$, the input gate $i_{t}$, the output gate $o_{t}$, the memory state $c_{t}$, and hidden state $h_{t}$ are as follows:

(4) $$\left\{\begin{array}{l} f_{t}=sigmoid\left(W_{f}⊗{\left[x_{t},h_{t-1}\right]}^{T}\right)\\ \;i_{t}=sigmoid\left(W_{i}⊗{\left[x_{t},h_{t-1}\right]}^{T}\right)\\ \;o_{t}=sigmoid\left(W_{o}⊗{\left[x_{t},h_{t-1}\right]}^{T}\right)\\ \;c_{t}=f_{t}⊗c_{t-1}⊕i_{t}⊗\tanh\left(W_{c}⊗{\left[x_{t},h_{t-1}\right]}^{T}\right)\\ \;h_{t}=o_{t}⊗tanh\left(c_{t}\right) \end{array}\right.$$

where sigmoid and tanh are the activation functions, $W_{\left(\cdot \right)}$ is the weight matrix, and $\left[x_{t},h_{t-1}\right]$ is the splicing vector between the input $x_{t}$ at moment t and the hidden state $h_{t-1}$ at the previous moment.

The forget gate of LSTM is used to control the forget strategy of $c_{t-1}$. The input gate and memory gate control the memory strategy of $x_{t}$ and $h_{t-1}$, respectively. Each LSTM cell outputs two important states: the memory state ($c_{t}$) and the hidden state ($h_{t}$). The information passed down from the previous time step $c_{t-1}$ is selected for forgetting and updating through the gate mechanism, thus maintaining the memory state of $c_{t}$ at time t. $c_{t}$ not only contains the information of the input at time t in the cell, but also contains historical information from previous time steps, and it is directly output to the next time step without further processing. For each time step, the changes to $c_{t}$ are relatively small. $h_{t}$ selects a portion of the content from $c_{t}$ as the output of the cell at time t through the output gate $o_{t}$. Compared to $c_{t}$, $h_{t}$ can focus more on the historical time information that is more important for the input at time t, that is, $h_{t}$ highlights the feature representation of the current moment more prominently. The differences in the $h_{t}$ passed to the next time step for inputs at different time steps are more noticeable.

Each Doppler feature sequence is fed into the LSTM network as the input to the corresponding LSTM cell. Typically, HAR networks use LSTM outputs in two ways: the first uses only the last time step output $h_{T}$, while the second uses all time step outputs $h_{i}\left(i=1,\ldots ,T\right)$. In the first case, as the hidden state with the longest time step, $h_{T}$ is believed to contain the temporal correlation information of the entire input sequences. However, since $h_{T}$ emphasizes the current time step’s feature expression, it may not “remember” relatively distant information, such as $h_{1}$. Therefore, obtaining accurate and reasonable feature expressions solely through $h_{T}$ is difficult. In the second case, outputting all time steps $h_{i}$ can ensure that the feature information at each time step can be fully utilized, but it also introduces irrelevant features that are not important for the current time. Both methods limit the recognition accuracy of HAR.

**Figure 9:**
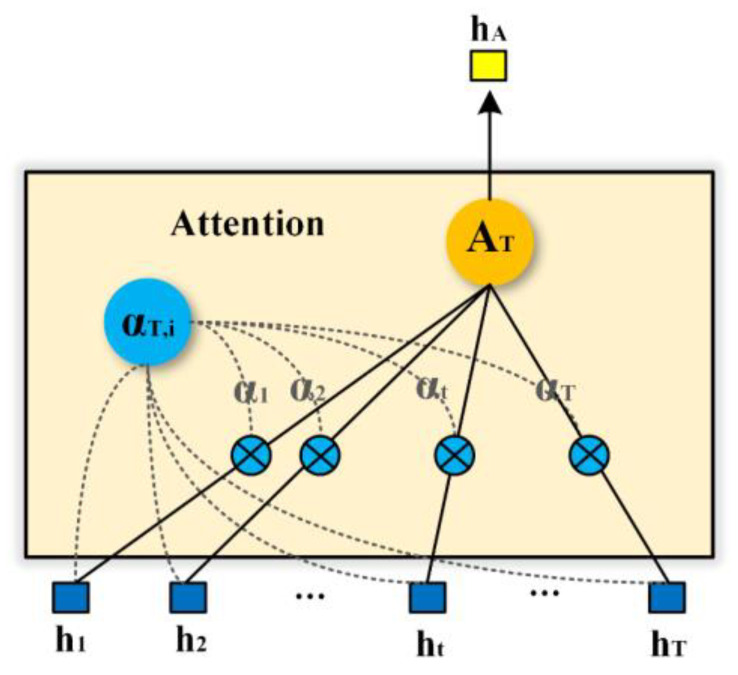
Network structure of the attention mechanism.

The proposed network combines these two methods using the attention mechanism. The attention mechanism takes full advantage of all the intermediate hidden layer outputs of the LSTM network, evaluates the importance of hidden layer information at every time step in combination with $h_{T}$ and correlates it with the outputs. The weight allocation mechanism in the attention mechanism focuses on important hidden layer temporal information, that is, the more important temporal features in the Doppler feature sequences. Therefore, the recognition accuracy of the hybrid network can be effectively improved by introducing the attention mechanism. The network structure of the attention mechanism is shown in Figure 9.

First, the weight of the output of each LTSM cell hidden layer is calculated. Correlation is performed to determine the similarity score between the output of each hidden layer and the final output.

(5) $$S_{T,i}=h_{T}{}^{T}h_{i}$$

Subsequently, the attention weights of each hidden layer $\alpha_{T,i}$ are obtained by normalizing $S_{T,i}$ using Softmax.

(6) $$\alpha_{T,i}=\frac{\exp\left(S_{T,i}\right)}{\sum_{k}\exp\left(S_{T,k}\right)}$$

Weighting $\alpha_{T,i}$ with each $h_{i}$ yields the weighting factor $A_{T}$.

(7) $$A_{T}=\sum_{i=1}^{T}\alpha_{T,i}h_{i}$$

$A_{T}$ provides the identification of the importance level for the features at different time steps. The final feature expression $h_{A}$ is the output of $A_{T}$, which can help the subsequent classification module make accurate judgments based on the feature importance. 

The final classification requires a transformation from $h_{A}$ to conditional probability distributions of different activity classes.

(8) $$P=softmax\left(W_{h}h_{A}+b_{h}\right)$$

where $W_{h}$ is the weight matrix and $b_{h}$ is the bias vector. Each element of P denotes the predicted probability of the p-th type of behavior.

During the training phase, cross-entropy is applied as the loss function. The label Y of the real activity is one-hot encoded, and the length Q is the number of activity categories. Cross-entropy is defined as the difference between the real activity label and the predicted action probability.

(9) $$loss=-\sum_{q=1}^{Q}Y_{q}\log\left(P_{q}\right)$$

Finally, the network parameters are continuously updated via backpropagation through time to reduce the loss value. Thus, the predicted value of the network converges to the real value.

## 4. Results and Discussions

### 4.1. Experimental Platform and Parameter Setting

**Figure 10:**
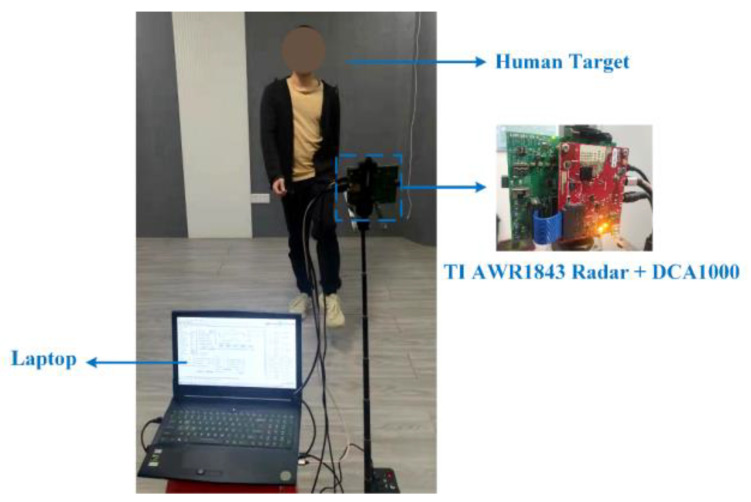
Experimental scenarios for collecting human activity data.

The experimental data were collected using TI’s radar platform at Guangzhou University in September 2021. The radar platform is composed of AWR1843 and DCA1000EVM. AWR1843 is a multi-channel millimeter-wave radar sensor, while DCA1000EVM is a capture card for interfacing with AWR1843 that enables users to stream digital IF data over the Ethernet to the laptop. The experiments were performed in an indoor environment with the radar platform mounted at a height of 1.5 m, as shown in Figure 10. The radar parameters are listed in Table 1.

In each individual activity, the IF signals were processed to generate a Doppler sequence group, containing 112 Doppler sequences of length 112. Sequentially, this group was fed into the subsequent DL networks for recognition. MATLAB was used for radar signal processing. Tensorflow v1.4.0 was used as the DL framework. All the networks were trained for 50 epochs using the adaptive moment estimation (Adam) optimizer with the batch size set to 64 and the learning rate set to 0.0001.

### 4.2. Dataset

In this study, we explored the optimal structure of the proposed network and completed the ablation experiment through a self-established dataset with five types of human behavior. The self-established dataset selected relatively simple activity acquisition patterns, and we will supplement more diverse and larger-scale activity patterns in our future work. Meanwhile, we conducted a comparative experiment to verify the superiority and adaptability of the proposed algorithm through a self-established dataset and a public dataset [30].

**Figure 11:**
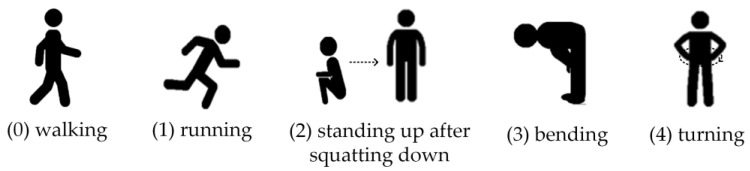
The sketch maps of the five human activities.

The self-established dataset records five human activities from 10 participants: (0) walking, (1) running, (2) standing up after squatting down, (3) bending, and (4) turning. Figure 11 shows the sketch maps of the five human activities. The range of movement of walking and running is about 1 m. Considering that the complete cycle of actions such as standing up after squatting down takes a relatively long time, the duration of each sample data is 5 s. The participants were seven males and three females with different heights, weights, and ages. During the experiment, for the diversity of the dataset, each participant acted according to their personal habits, without constraints on specific activities. Particularly, each participant repeated a specific activity 20 times. Totally, the activity distribution generated 1000 Doppler sequence groups. The public dataset developed by the University of Glasgow, UK, contains six human activities: walking, sitting, standing, picking up an object, drinking water, and falling down. Among them, the duration of walking is 10 s, and the duration of the other activities is 5 s. The range of movement of walking is about 60 m. They employed an FMCW radar with a 5.8 GHz operating frequency and a chirp bandwidth of 400 MHz to record the data. A total of 1754 micro-Doppler signature samples were generated. The datasets were randomly divided into 60% for training, 20% for validating, and 20% for testing.

### 4.3. Discussion of the Proposed Network Structure

The specific recognition performance and the efficiency are closely related to the network structure. In the proposed CLA hybrid network, depth (the number of layers in a neural network) and width (the number of channels in one layer) are often used to describe the network hierarchy of the 1D CNN, which reflects the ability to conceptualize Doppler features from the input Doppler sequences. 

The number of LSTM CELLs in a single-layer LTSM network is determined by the number of timing states required for HAR. Herein, the CELL number is fixed as 112. This value is closely related to the signal acquisition duration and Doppler window interval, and its optimization is not within the scope of this study. The hidden state dimension in the LSTM CELL decides the expressiveness of the network under the current time. With the increasing number of hidden units, more feature details are contained, and the expression ability is richer; however, it causes parameters surging and is time consuming.

The hybrid network CLA requires a trade-off between the time consumption and the expression ability. The feature representative capability can be amplified during training due to the positive feedback between layers. Deepening of the network can improve the nonlinear expression ability, which can implement more complex feature fitting. The width of each layer, that is, the number of convolutional kernels, determines the richness of the captured features in each layer, which is correlated to the difficulty of network optimization. However, blindly deepening the network will cause optimization difficulties, performance saturation, and degradation of the shallow learning capability. Similarly, exceeding the appropriate width will reduce the network efficiency due to repeated feature extraction.

Therefore, to determine the optimal structure of the proposed CLA network, the depth, and width of the 1D CNN as well as the number of LSTM hidden units were verified to analyze the corresponding network performance. The parameter quantity, the inference time, and the accuracy are the indicators of the network performance. Since the frequency domain representation is not sparse and the kernel size is not a key factor affecting the accuracy, CLA adopts a fixed 3-length convolution kernel for 1D CNN. The time step of LSTM is fixed at 112, which also fixes the time series feature number of the connections in the subsequent attention module. All structures were analyzed under the condition of Epochs = 50. The experimental results are listed in Table 2.

Table 2 shows that the improvement of accuracy is basically consistent with the increase in network complexity. Moreover, the network performance gradually saturates as the depth of the 1D CNN expands. Specifically, the accuracy of the CLA network with 64–128 1D CNN and 32 LSTM hidden units is close to that of the CLA network with 64–128–256 1D CNN and 32 LSTM hidden units, but the amount of network parameters of the latter is nearly twice that of the former. 

In terms of the network efficiency, when the two basic dimensions (depth and width) of 1D CNN are fixed, the accuracy increases with the increase in LSTM hidden units, but the parameters and inference time also increase. Moreover, when the LSTM hidden units reach a certain level, the network also gradually saturates. From Table 2, the accuracy of the CLA network with 64–128 1D CNN and 128 LSTM hidden units only has a slight increase compared with the CLA network with 64–128 1D CNN and 64 LSTM hidden units, but the network parameters and inference time of the former were nearly twice that of the latter.

Finally, the CLA network based on 64–128 1D CNN and 32 LSTM hidden units, marked in bold in Table 2, is analyzed, compared, and discussed in the following sections. It has the smallest number of parameters and the shortest inference time among the networks, with nearly 97% accuracy.

### 4.4. Ablation Experiments

**Figure 12:**
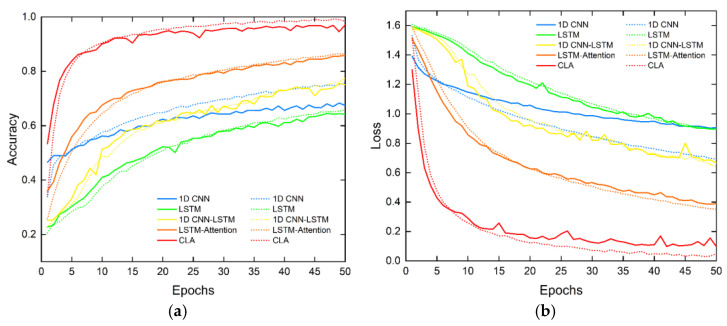
(**a**) Curves of training and validating accuracy varying with the number of epochs for different networks in ablation experiments; (**b**) curves of training and validating loss varying with the number of epochs for different networks in ablation experiments. (The solid line represents the validating curve; the dotted line represents the training curve).

**Figure 13:**
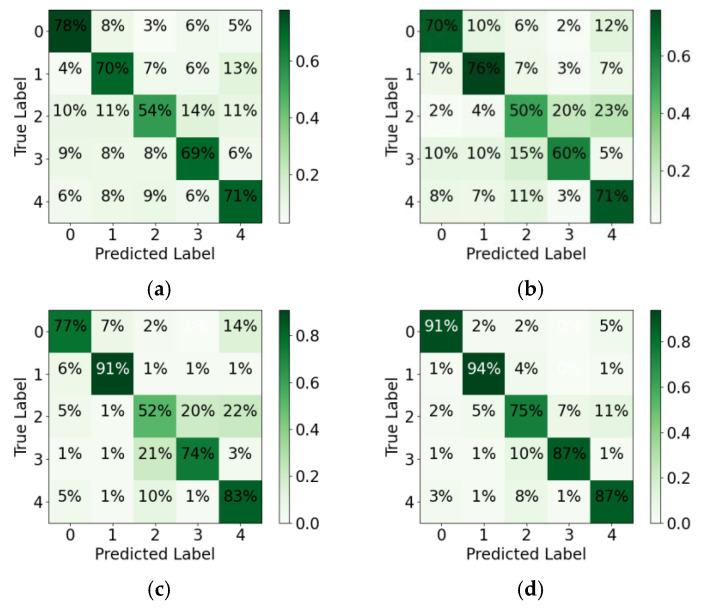

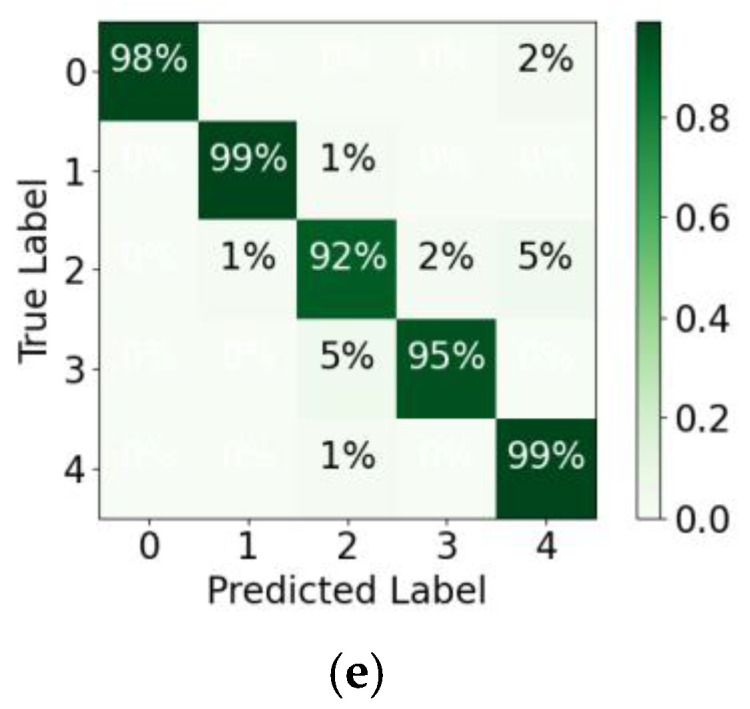
Confusion matrices for different networks in ablation experiments: (**a**) 1D CNN; (**b**) LSTM; (**c**) 1D CNN-LSTM; (**d**) LSTM-Attention; (**e**) CLA.

This section discusses ablation to evaluate each module’s contribution to the proposed CLA network. The change in accuracy is examined when removing different modules. In total, five network structures are discussed: 1D CNN, LSTM, 1D CNN-LSTM, LSTM-Attention, and CLA. The detailed training, validating, and testing results are shown in Figure 12 and Figure 13 and Table 3.

The comparison shows that the attention-based hybrid network is superior to the single networks in terms of network performance. From the perspective of the training and validating process, single networks, such as 1D CNN and LSTM, have a weak ability to represent the HAR features due to their limited network structures. Their accuracy and convergence speed are lower than those of the hybrid network. In addition, for 1D CNN, there be some differences between the training and validating performance.

The hybrid networks comprising two networks were compared. The results show that the hybrid networks all outperform the single network, and the 1D CNN-LSTM with poorer performance in the hybrid network has 7.93% higher accuracy than the 1D CNN with better performance in the single network. The performance of LSTM-Attention is closer to CLA in terms of the indicators and convergence curves. Its accuracy is higher than that of 1D CNN-LSTM and single LSTM networks by 10.02% and 19.92%, respectively. Unlike LSTM, the attention mechanism performs a weighted synthesis of all the hidden states output using the LSTM module at different time steps; this effectively improves the HAR network performance. Furthermore, this result implies that temporal features embedded in the radar signals have more explicit representations of different human activities than Doppler features.

The CLA network performs significantly better than the other four networks. It requires the fewest epochs and its network converges the fastest. Among the five networks, the CLA network affords the highest accuracy and the least inference time. On the basis of LSTM-Attention, the CLA applies 1D CNN to pre-extract the Doppler features of the input sequences, effectively solving the insufficient representation of Doppler features by LSTM-Attention. Consequently, the recognition accuracy is improved by 11.06%.

The results of the confusion matrices show that the actions of label 2 (squatting and standing) and label 3 (bending) are easier to confuse than those of other labels. Since HAR is performed depending on the time-dependent changes of the micro-Doppler components introduced by the limb movements, the frequency characteristics of the limb movements determine how similar the actions are. For label 0 (walking), its time-varying frequency is similar to that of label 4 (turning), so there is a misclassification between them. Label 1 (running) has a high recognition probability due to its high movement frequency. For labels 2, 3, and 4, the symmetry, variation law, and amplitude of their micro-Doppler distribution are similar in some cases; thus, they exhibit similar features along slow time in the Doppler domain, leading to recognition errors. However, since CLA integrates Doppler features and attention-based weighted time series features, the recognition accuracy can still be greatly improved.

### 4.5. Attention Mechanism Discussion

**Figure 14:**
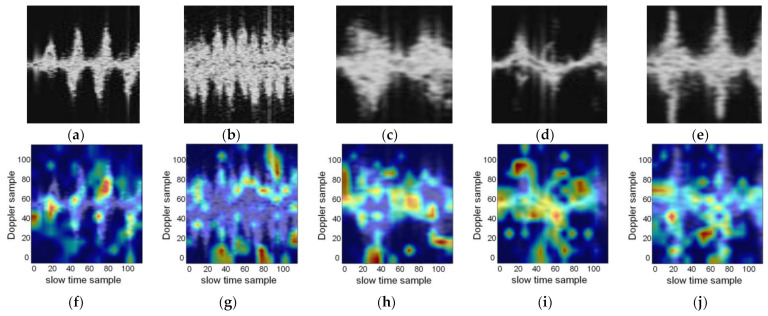
The MDM and feature heatmap of the attention mechanism for the different activities: (**a**) grayscale MDM of label 0; (**b**) grayscale MDM of label 1; (**c**) grayscale MDM of label 2; (**d**) grayscale MDM of label 3; (**e**) grayscale MDM of label 4; (**f**) heatmap of label 0; (**g**) heatmap of label 1; (**h**) heatmap of label 2; (**i**) heatmap of label 3; (**j**) heatmap of label 4.

To obtain a detailed understanding of the role of the attention mechanism in the HAR task based on the radar signals, the part of the features that the attention mechanism focuses on is visualized. A heatmap can help obtain a visual representation from the integrated MDM by highlighting the regions considered to be important for HAR. The first row of Figure 14 displays the grayscale MDM images of the five labeled activities. These images are formed by arranging the input Doppler sequences in the time series. The heatmaps of the grayscale MDM images above are correspondingly displayed in the second row. The red parts in the heatmaps denote the regions that the network focuses on. The attention heatmaps clearly show that most of the red regions are distributed in the endpoint and contour positions that reflect the change of the micro-Doppler distribution. These concerns are consistent with the Doppler distribution characteristics of different activities in the radar signals. Moreover, this demonstrates that CLA can focus on the more representative features in the input sequences along the slow time. Therefore, the attention mechanism improves the performance of CLA.

### 4.6. Comparison of Different Literature Networks

**Figure 15:**
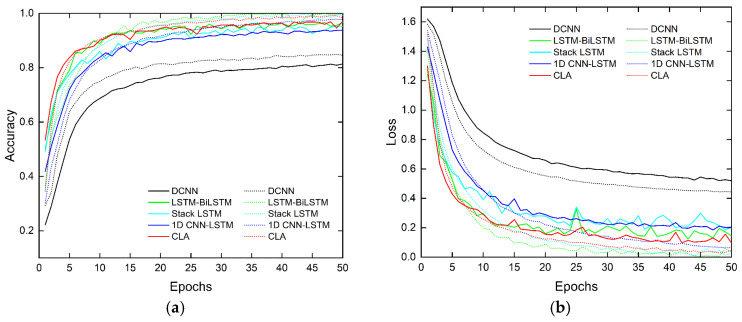
(**a**) Curves of training and validating accuracy varying with the number of epochs for the literature networks; (**b**) curves of training and validating loss varying with the number of epochs for the literature networks. (The solid line represents the validating curve; the dotted line represents the training curve).

In this section, multiple networks from the literature are employed for performance comparison. The parameters, inference time, and floating point operations (FLOPs), which indicate the possibility of embedded applications of the algorithm, are taken into consideration while discussing the accuracy. The parameters, inference time, and FLOPs are important indicators that reflect the complexity of a network. Larger parameters and higher FLOPs usually result in a longer inference time, but this is not absolute and is also related to factors such as network structure and computational device performance. To fully evaluate the speed and efficiency of different network models, these three indicators need to be considered comprehensively. Table 4 summarizes the results of these state-of-the-art studies in terms of HAR with the self-established dataset. The training and validating of the networks results are shown in Figure 15.

The feature extraction method based on 2D images was adopted in [5]. The researchers treated the output MDM of STFT as a 2D image and used a CNN with a 2-layer of 5 × 5 convolutional kernels to extract the local spatial features. Although this network has few parameters and a simple structure, it does not explore the difference between MDM feature expression and visual images. Thus, the HAR accuracy rate is only 81.03%, and the inference time is 0.94 ms. 

Considering the timing characteristics of HAR in the radar signals, the literature [12,13] achieved high recognition accuracy and fast convergence using an LSTM-BiLSTM hybrid network and a stacked LSTM network with three layers, respectively. The HAR accuracy of both networks is higher than 95%, but the results in Table 4 denote that the high accuracy of [12,13] comes at the cost of a large number of network parameters and time overhead. In [12], the number of parameters reached 1.5 M, FLOPs reached 2.9 M, and the inference time of a single sample reached 5.12 ms. This is attributed to the serial network structure between the LSTM CELLs and the fully connected structure in each LSTM CELL. Multi-layer stacking of LSTMs or the combination of LSTM and BiLSTM will lead to a significant increase in the network size. Moreover, LSTM relies on the calculation of the previous moment to obtain the results of the next moment; therefore, FLOPs and the inference time will increase when more LSTM CELLs are introduced. In addition, combining the curves in Figure 15, we can see that the networks of [12,13] have different degrees of overfitting problems. One reason is that the number of parameters is too large, and the fitting ability of the neural network becomes very strong, which means that the expressed function will be more complex, and it is very easy to cause overfitting by using overly complex functions. The second reason is that the dropout is applied in the training process, but not in the validation process, which can also lead to overfitting; however, based on the second reason, the overfitting problem of CLA is obviously less serious under the same conditions. The network comparison with [17] demonstrates the superiority of the attention mechanism due to the focus on the key time features. The proposed CLA network can achieve an accuracy improvement of 2.92% and a faster convergence speed compared to [17]. Moreover, the proposed CLA has higher accuracy and lower network complexity compared to the previous literature networks. In addition, we also used the public dataset to compare the accuracy of different literature networks (Accuracy* in Table 4); the results are consistent with the above discussion.

### 4.7. Clutter Suppression Performance Experiment

**Figure 16:**
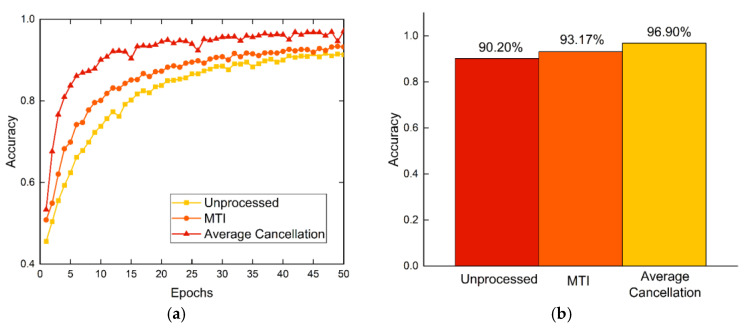
Recognition performance of three clutter suppression algorithms: (**a**) curves of validating accuracy varying with the number of epochs and (**b**) test accuracy.

To verify the superiority of the average cancellation method used herein for improving the recognition performance in HAR, two other datasets were established with the collected radar digital IF data. The two datasets were processed without clutter suppression and with MTI. The proposed CLA is applied on these three datasets and the results are shown in Figure 16.

The comparison shows that the dataset processed by the average cancellation method yields higher accuracy using the same recognition network than the other two datasets. The accuracy is higher by 6.70% and 3.73% compared to the dataset without clutter suppression and the MTI processing dataset, respectively. This is mainly because the average cancellation method effectively suppresses the static clutter interference in micro-Doppler application. Additionally, it preserves and highlights the MDFs introduced by human activities more completely. The experimental results fully reveal that to realize the performance improvement of HAR, the features of human activity reflected in radar signals need to be fully utilized and deeply mined from both radar preprocessing and DL network optimization.

## 5. Conclusion

This paper focuses on the precise and fast human activity recognition problem. We combined radar sensors with a DL network and proposed a HAR solution with an Attention-based CLA network. The solution includes two parts: radar signal preprocessing and a DL network. In the radar preprocessing part, the average cancellation method is used to replace the MTI algorithm, which effectively realizes static clutter suppression under micro-Doppler conditions and improves the accuracy of HAR. The CLA fully exploits the Doppler and timing characteristics of the target activities reflected in the radar signal, which uses 1D CNN+LSTM joint modeling to effectively extract temporal features. Attention can further enhance the ability of feature extraction, enabling the network to better understand important features. CLA adopts a relatively lightweight network structure, which can achieve higher recognition accuracy. In the experiment, structure optimization, an ablation experiment, and an attention mechanism discussion were conducted for the proposed CLA based on a self-established dataset. Comparative experiments with existing methods were conducted on two datasets (self-established and public), confirming the superiority of CLA in performance and efficiency. Therefore, the proposed solution can meet the requirements of accuracy and low complexity for HAR and has great potential for real-time embedded applications. This article only recognized individual actions, and the verification of complex and continuous actions is not yet sufficient. In the future, we plan to improve and expand the categories and quantities of the dataset and use multi-level feature extraction and reinforced DL methods to further improve recognition performance.

## Figures and Tables

**Table 1:** FMCW radar parameters.

Parameters	Value
Carrier frequency (*f_c_*)	77 GHz
Bandwidth (*B*)	3680 MHz
Chirp duration time (*T*)	392 us
Frame repetition time (*T_F_*)	100 ms
Sampling frequency (*f_s_*)	4 MHz
Sampling number (*N*)	128
Number of chirp (*L*))	255
Number of frame (*N_F_*)	50
Number of transmit channels	1
Number of receive channels	4

**Table 2:** Discussion of CLA network structure.

Depth of CNN	Width of CNN	LSTM Hidden Units	Parameters	Inference Time /Sample (ms)	Accuracy (%)
1	16	32	42,245	0.32	89.64
		64	74,629	0.49	90.12
		128	163,973	0.87	92.12
	32	32	49,685	0.32	92.72
		64	84,117	0.50	93.12
		128	177,557	0.89	95.01
	64	32	64,565	0.33	95.12
		64	103,093	0.50	95.98
		128	204,725	0.90	96.13
**2**	16–32	32	45,861	0.32	91.02
		64	80,293	0.46	91.68
		128	173,733	0.88	92.32
	32–64	32	59,989	0.34	94.15
		64	98,517	0.50	95.39
		128	200,149	0.90	95.74
	**64–128**	**32**	**97,461**	**0.43**	**96.90**
		64	144,181	0.58	97.17
		128	262,197	0.99	97.46
3	16–32–64	32	56,165	0.34	92.39
		64	94,693	0.50	94.23
		128	196,325	0.93	94.39
	32–64–128	32	92,885	0.44	96.44
		64	139,605	0.60	96.46
		128	257,621	1.02	96.73
	64–128–256	32	212,405	0.64	97.49
		64	275,509	0.85	97.51
		128	426,293	1.24	97.78
4	16–32–64–128	32	89,061	0.44	94.42
		64	135,781	0.62	94.58
		128	253,797	1.05	94.63

**Table 3:** Performance comparison of ablation experiments.

Network	Parameters	Inference Time /Sample (ms)	Accuracy (%)
1D CNN	117,957	0.13	67.89
LSTM	18,725	0.26	65.92
1D CNN-LSTM	67,045	0.36	75.82
LSTM-Attention	49,141	0.32	85.84
CLA	97,461	0.43	96.90

**Table 4:** Performance comparison of the literature networks.

Network	Parameters	Inference Time/Sample (ms)	FLOPs	Accuracy (%)	Accuracy * (%)
DCNN [5]	4433	0.94	4457	81.03	91.01
LSTM-BiLSTM [12]	1,531,205	5.12	2,910,728	96.22	96.41
Stack3-LSTM [13]	458,245	2.32	849,928	95.29	95.67
1D CNN-LSTM [17]	93,029	0.44	125,448	93.98	94.16
CLA	97,461	0.43	105,225	96.90	96.94

* indicates the accuracy on a public dataset.

## Data Availability

The data presented in this study are available on request from the corresponding author.
